# Trojan‐Horse Strategy Targeting the Gut‐Liver Axis Modulates Gut Microbiome and Reshapes Microenvironment for Orthotopic Hepatocellular Carcinoma Therapy

**DOI:** 10.1002/advs.202310002

**Published:** 2024-10-07

**Authors:** Haochen Yao, Sheng Ma, Juanjuan Huang, Xinghui Si, Ming Yang, Wantong Song, Guoyue Lv, Guoqing Wang

**Affiliations:** ^1^ Hepatobiliary and Pancreatic Surgery Department General Surgery Center First Hospital of Jilin University No.1 Xinmin Street Changchun Jilin 130021 China; ^2^ Key Laboratory of Zoonosis Chinese Ministry of Education College of Basic Medical Sciences Jilin University Changchun Jilin 130021 China; ^3^ Key Laboratory of Polymer Ecomaterials Changchun Institute of Applied Chemistry Chinese Academy of Sciences 5625 Renmin Road Changchun 130022 China; ^4^ Jilin Biomedical Polymers Engineering Laboratory Changchun Institute of Applied Chemistry 5625 Renmin Road Changchun 130022 China; ^5^ Department of Computational Mathematics School of Mathematics Jilin University Changchun 130012 China; ^6^ Department of Molecular Biology College of Basic Medical Sciences Jilin University Changchun 130021 China

**Keywords:** gut microbiota, hepatic inflammation, hepatocellular carcinoma, immunotherapy, oral nanomedicine

## Abstract

Reversing the hepatic inflammatory and immunosuppressive microenvironment caused by gut microbiota‐derived lipopolysaccharides (LPS), accumulating to the liver through the gut‐liver axis, is crucial for suppressing hepatocellular carcinoma (HCC) and metastasis. However, synergistically manipulating LPS‐induced inflammation and gut microbiota remains a daunting task. Herein, a Trojan‐horse strategy is proposed using an oral dextran‐carbenoxolone (DEX‐CBX) conjugate, which combines prebiotic and glycyrrhetinic acid (GA) homologs, to targeted delivery GA to HCC through the gut‐liver axis for simultaneous modulation of hepatic inflammation and gut microbiota. In the orthotopic HCC model, a 95–45% reduction in the relative abundances of LPS‐associated microbiota is observed, especially *Helicobacter*, caused by DEX‐CBX treatment over phosphate‐buffered saline (PBS) treatment. Notably, a dramatic increase (37‐fold over PBS) in the abundance of *Akkermansia*, which is known to strengthen systemic immune response, is detected. Furthermore, DEX‐CBX significantly increased natural killer T cells (5.7‐fold) and CD8^+^ T cells (3.9‐fold) as well as decreased M2 macrophages (59% reduction) over PBS treatment, resulting in a tumor suppression rate of 85.4%. DEX‐CBX is anticipated to offer a novel strategy to precisely modulate hepatic inflammation and the gut microbiota to address both the symptoms and root causes of LPS‐induced immunosuppression in HCC.

## Graphical abstract

This study elucidates a new strategy to address both the symptoms and root cause of gut microbiota‐derived substance‐induced immunosuppression for HCC immunotherapy by precisely modulating hepatic inflammation and gut microbiota with oral nanomedicine.

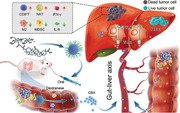

## 1 Introduction

Hepatocellular carcinoma (HCC) is the third leading cause of cancer‐related deaths worldwide^[^ ^1^ ^]^ and is a major complication of end‐stage liver fibrosis and cirrhosis. As the liver functions as a detoxification organ,^[^ ^2^ ^]^ the liver exhibits strong intrinsic immune tolerance.^[^ ^3^ ^]^ Additionally, HCC induces the enhanced permeability of intestinal mucosal and leads to a high level of lipopolysaccharides (LPS) in the hepatic portal vein and peripheral vein, causing continuous hepatic inflammation and immunosuppression.^[^ ^4^ ^]^ Immunotherapy, especially immune checkpoint inhibitors such as PD‐1/PD‐L1 antibodies, has made only limited progress in HCC clinical management.^[^ ^5^ ^]^ Emerging evidence suggests that gut bacterial products and translocation are critical in hepatocarcinogenesis and HCC cell proliferation by activating Toll‐like receptor 4 (TLR4) on macrophages with LPS, both directly and indirectly. LPS can directly activate TLR4 assisted by MD‐2. Additionally, LPS can trigger increased release of high mobility group box 1 (HMGB1) from hepatocytes, which also acts as a ligand for TLR4, thereby indirectly activating TLR4. Therefore, LPS and gut microbiota represent potential therapeutic targets for HCC treatment.

Glycyrrhetinic acid (GA), the major bioactive hydrolysis product of glycyrrhizic acid (GL),^[^ ^6^ ^]^ possesses remarkable modulatory capability in TLR4‐associated inflammation. The pentacyclic triterpenoid structure along with the carbonyl group at position 11 and the carboxyl group at position 30 in GL, which are the major structural features of GA homologs, have been comfired to be the key structures for their biological activity.^[^ ^7^ ^]^ GA and GA homologs including GL and carbenoxolone (CBX) exhibit potent inhibition in LPS‐induced HMGB1 selection from macrophages and hepatocytes, thereby bolstering the suppression of TLR4‐associated inflammation.^[^ ^8^ ^]^ Moreover, GA homologs have been shown to disrupt the formation of the LPS/TLR4/MD‐2 complex,^[^ ^9^ ^]^ as well as inhibit downstream NF‐κB and MAPK activation by reacting with the cysteines in TLR4, resulting in suppression of LPS‐induced TLR4 activation. However, the intense side effects and the low bioavailability of GA and GA homologs hinder their application in HCC treatment. Targeted and efficient delivery of GA homologs to HCC tissues is essential to maximize their therapeutic potential.

Nanotechnology‐based drug delivery systems are powerful tools for improving the biodistribution and bioavailability of drugs.^[^ ^10^ ^]^ Controlled‐release drug delivery systems, in particular, can enhance the accumulation of active drugs at desired sites.^[^ ^11^ ^]^ Several studies have attempted to construct nanoformulations of GA homologs to enhance their in vivo therapeutic performance.^[^ ^12^ ^]^ However, the therapeutic progress achieved with these combinations of GA and chemotherapeutics has been limited, largely due to overlooking the intricate interactions between gut microbiota and HCC.^[^ ^13^ ^]^ These nanomedicines have typically been administered intravenously, while, oral administration holds greater promise due to its convenience and non‐invasiveness. Moreover, oral administration may be particularly advantageous for triggering drug activation and improving bioavailability, especially with the presence of specific metabolic enzymes such as dextranase in the colon.^[^ ^14^ ^]^ This makes oral administration a competitive candidate for enhancing the therapeutic efficacy of GA homologs in HCC treatment.

**Scheme 1:**
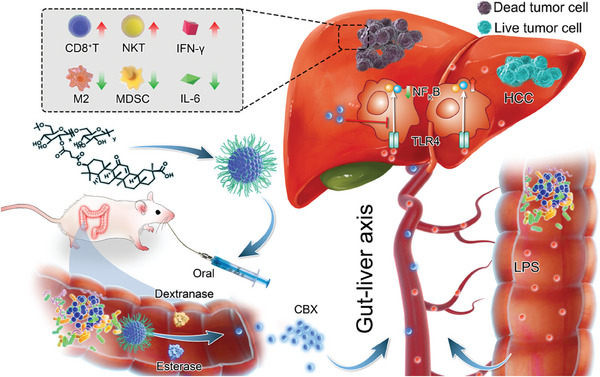
Schematic illustration of the mechanism of orally administered DEX‐CBX for HCC therapy. GA can be released from orally administrated DEX‐CBX inside the colon in the presence of gut bacterial secreted dextranase and esterase, and suppressing LPS‐mediated TLR4 activation after arriving at HCC through the gut‐liver axis, which results in relieving of tumor immunosuppressive TME, characterized by reduced content of M2 macrophages, MDSCs and IL‐6, and increased content of CD8^+^ T cells, NKT cells and IFN‐γ. The structures of CBX, GA, and GL are resented in Figure S1 (Supporting Information).

Here, we propose a Trojan‐horse strategy with an oral dextran‐carbenoxolone (DEX‐CBX) conjugate, which leverages colon‐specific enzymes, the gut‐liver axis, and prebiotics (**Scheme** **1**). This DEX‐CBX conjugate acts as a Trojan horse, safeguarding the drug from degradation by gastric juice and absorption in the small intestine. Upon reaching the colon, GA is released from DEX‐CBX in the presence of dextranase and esterase. The cascaded action of gut microbiota‐derived enzymes triggering drug activation and targeted accumulation via the gut‐liver axis provides a novel and non‐invasive GA delivery pathway to HCC, thereby enhancing inflammation relief and modulating the tumor immune microenvironment. Furthermore, dextran cooperated with hepatic inflammation relief to reduce the abundance of harmful gut commensal species and increase probiotics. This study sheds light on the impact of utilizing oral nanomedicine for simultaneous regulation of the HCC immune microenvironment and the gut commercial microbiota in HCC immunotherapy. The synergistic effect of gut microbiota‐derived enzymes triggering drug activation, alongside targeted accumulation via the gut‐liver axis, offers a novel solution for the precise delivery of immune modulators to HCC tissue.

## 2 Results and Discussion

### 2.1 Synthesis and Characterization of DEX‐CBX

**Figure 1:**
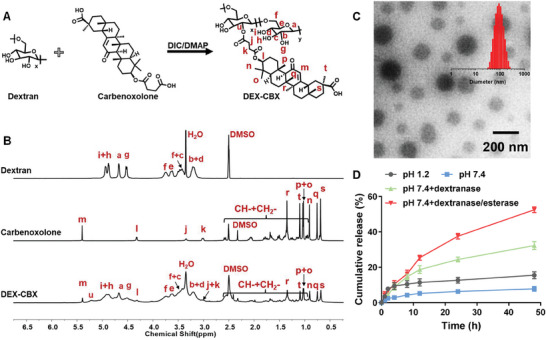
Synthesis and solution behavior of DEX‐CBX. A) Synthesis routes of DEX‐CBX. B) ^1^H NMR spectrum of DEX, Carbenoxolone, and DEX‐CBX in DMSO‐d_6_. C) Representative TEM image and hydrodynamic diameters of DEX‐CBX measured by DLS. D) In vitro GA release profiles of DEX‐CBX in buffer containing 0.2% (w/v) Tween 80 at different conditions: pH 1.2, pH 7.4, pH 7.4 with dextranase and pH 7.4 with dextranase and esterase. Data are shown as means ± SD (n = 3).

DEX, a bacterial‐derived polysaccharide, exhibits excellent biocompatibility and can be enzymatically hydrolyzed into monosaccharides for site‐controllable drug release by colon‐specific dextranase.^[^ ^15^ ^]^ More importantly, prebiotics, including DEX, have shown considerable potential in modulating the gut microbiota for colorectal cancer management.^[^ ^16^ ^]^ Based on the above facts, DEX was selected as the oral delivery carrier. DEX‐CBX was synthesized by directly conjugating CBX with DEX via esterification (**Figure** **1**A). ^1^H NMR spectrum confirmed the successful synthesis of DEX‐CBXs. As shown in Figure 1B, the characteristic hydrogen peaks of the C‐C double bonds on adjacent carbonyl carbon of CBX (‐COCH‐, m, 5.40 ppm) and alkyl carbon (‐CH3, n/o/p/q/r/s/t, 1.02 ppm/1.03 ppm/0.75 ppm/1.35 ppm/0.68 ppm/1.09 ppm), appeared in the ^1^H NMR spectrum of DEX‐CBX. In addition, the chemical shifts of methine protons of glucose unit in DEX switched from 3.21 ppm (b) to 5.22 ppm (u) after esterification between CBX and DEX. There are two carboxyl groups in CBX, the ^13^C NMR spectrum confirmed the carboxyl group away from the triterpenoid skeleton (v) implemented the conjugation between CBX and DEX as the chemical shift of v completely switched from 173.16 to 172.17 ppm (Figure S2, Supporting Information). Furthermore, Fourier‐transform infrared (FT‐IR) results confirmed that the carboxyl groups in CBX participated in the synthesis of DEX‐CBX as a decrease of carboxyl peak at λ 1703 could be observed (Figure S3, Supporting Information). The gel permeation chromatography results confirmed that DEX‐CBX owned a larger molecular weight than DEX, the polydispersity index of DEX‐CBX was 1.79 (Figure S4, Supporting Information). These results prove that CBX was successfully conjugated to DEX. To accurately assess the drug loading content, we utilized high‐performance liquid chromatography (HPLC) to detect the hydrolysis products of DEX‐CBX treated with NaOH. Given that CBX can be converted to GA during NaOH hydrolysis, the content of GA in each hydrolysis solution was measured. The content of CBX was then calculated based on the molecular weight ratio between CBX and GA, revealing a drug‐loading content of CBX at 16.8% (Figure S5, Supporting Information).

Despite CBX has extremely strong hydrophobicity, DEX‐CBX could easily self‐assemble into nano micelles in PBS. Dynamic light scattering (DLS) measurements revealed a Z‐average diameter of 92.3 nm and a polydispersity index (PDI) of 0.10 for DEX‐CBX nanoparticles. The transmission electron microscope (TEM) image confirmed the spherical morphology of the DEX‐CBX assemblies with an average diameter were 94.4 ± 21.2 nm (Figure 1C). The size of DEX‐CBX maintained constant in PBS across 48 h (Figure S6, Supporting Information). Nanoassemblies should exhibit considerable resistance to the digestive fluids in the stomach. As expected, the release rates of DEX‐CBX at pH 1.2 NaCl–HCl buffer and pH 7.4 PBS were all slow, and the calculated release ratios were both below 15% in 48 h (Figure 1D). Dextranase hydrolyzed the glycosidic linkage of DEX, which fragmented DEX‐CBX and cooperated with the hydrolysis of DEX‐CBX.^[^ ^17^ ^]^ Thus, in the presence of dextranase, the release rate of DEX‐CBX was accelerated, resulting in ≈30% of total CBX released at pH 7.4 PBS with dextranase. Furthermore, under the condition of pH 7.4 PBS with dextranase, and esterase, the release rate of DEX‐CBX was further improved, and more than 50% of CBX was released from DEX‐CBX in 48 h, suggesting the feasibility of colonic enzymatic conditions to trigger the drug release of DEX‐CBX.

### 2.2 In Vitro Suppression of LPS‐Induced Inflammation and Tumor Cell Proliferation

**Figure 2:**
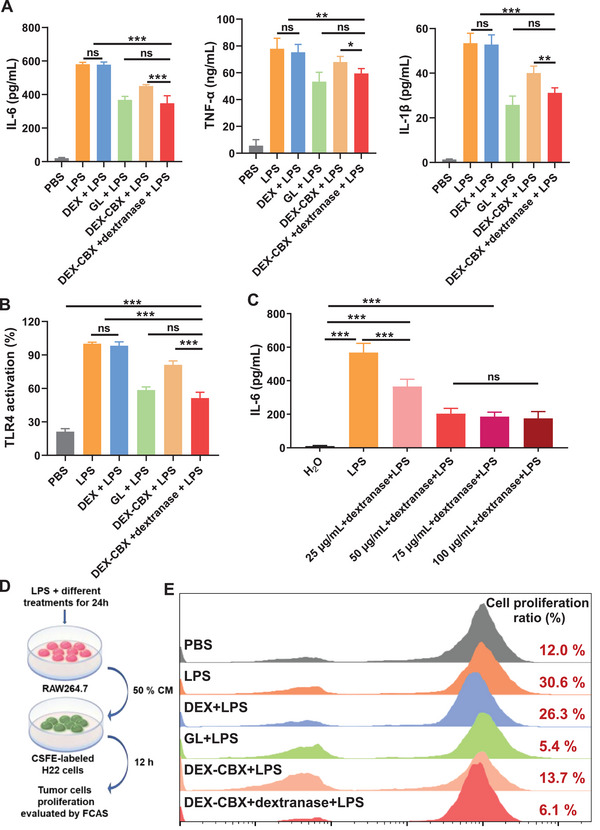
Suppression of LPS‐induced inflammation and tumor cell proliferation. A) The IL‐6, TNF‐α, and IL‐1β levels of RAW2.64.7 cells incubated with or without LPS after different treatments. B) The level of TLR4 activation of HEK‐Blue hTLR4 cells after different treatments. C) The IL‐6 levels of RAW2.64.7 cells incubated with LPS with different concentrations of DEX‐CBX. D) Schematic illustration of in vitro suppression of LPS‐induced tumor cells proliferation experiment. E) The flow cytometry results of in vitro suppression of LPS‐induced tumor cell proliferation. Data are shown as means ± SD (n = 3). ^*^ *p* < 0.05, ^**^ *p* < 0.01, ^***^ *p* < 0.001; ns, no significance.

Gut microbiota and related substances have been proposed to contribute to the progression of various hepatic diseases, including HCC, through various means, among which LPS of gut microbiota has been demonstrated to be a critical driver to facilitating HCC by activating TLR4. LPS can activate TLR4 signaling in resident liver cells, particularly Kupffer cells and hepatic stellate cells to stimulate the secretion of cytokines such as interleukin 6 (IL‐6) and tumor necrosis factor‐alpha (TNF‐α), leading to hepatic inflammation and oxidative damage.^[^ ^18^ ^]^ We first investigated the suppressive effects of DEX‐CBX on LPS‐mediated TLR4 activation. As shown in **Figure** **2**A, LPS treatment significantly elevated the secretion of inflammatory cytokines by RAW264.7 cells including IL‐6, TNF‐α, and interleukin‐1 beta (IL‐1β). The treatment of 36 µg mL^−1^ GL decreased the inflammatory cytokines level. Similar to GL treatment, the treatment of 25 µg CBX/mL of DEX‐CBX with dextranase as well as 25 µg CBX/mL of DEX‐CBX alone could attenuate LPS‐induced inflammatory cytokines level. In contrast, DEX treatment did not show this suppression ability. We further utilized HEK‐Blue hTLR4 cells to confirm that DEX‐CBX alleviated the release of inflammatory cytokines induced by LPS through suppression of TLR4 activation.^[^ ^19^ ^]^ HEK‐Blue hTLR cells were genetically co‐transfected with the hTLR4 gene, the MD‐2/CD14 coreceptor genes, and a secreted embryonic alkaline phosphatase (SEAP) reporter gene to study the activation of TLR4 by testing SEAP. As shown in Figure 2B, LPS induced dramatically elevated levels of SEAP compared with PBS, indicating the activation of TLR4 caused by LPS. The treatment of GL and DEX‐CBX with/without dextranase all could significantly block the activation of LPS on the TLR4 pathway. Particularly, DEX‐CBX with dextranase exhibited a similar inhibitory effect on TLR4 activation as free GL, whereas dextran alone did not demonstrate such an effect. Therefore, DEX‐CBX can modulate inflammatory responses caused by LPS via targeting the interaction between LPS and TLR4. Subsequently, we continue to investigate the correlation between the therapeutic concentration of DEX‐CBX and IL‐6 secretion on RAW264.7 cells. A much lower IL‐6 level was detected as the concentration of DEX‐CBX increased up to 50 µg mL^−1^ (Figure 2C). When the concentration of DEX‐CBX was over 50 µg mL^−1^, the IL‐6 secretion was not further reduced. IL‐6 promotes tumor cell proliferation in an inflammatory environment.^[^ ^20^ ^]^ Thus, we proposed that a reduction in IL‐6 levels is a powerful tool to inhibit HCC cell proliferation. To verify our hypothesis, we used the conditioned culture medium of RAW264.7, pretreated with PBS, LPS, or LPS combined with different treatments to culture CSFE‐labeled H22 cells (Figure 2D). As shown in Figure 2E, the top‐performing treatments for IL‐6 inhibition, including GL, DEX‐CBX, and DEX‐CBX along with dextranase, reduced LPS‐induced H22 cell proliferation. As the IC50 values of GL and DEX‐CBX to H22 cells (the IC50 of GL and DEX‐CBX to H22 cells at 24 and 48 h were 347.3, >500 and 265.7, >500 µg mL^−1^, respectively) were much higher than the dose used in this experiment (Figure S7, Supporting Information), thus, the tumor cells proliferation inhibition of GL and DEX‐CBX should mainly attribute to inflammation modulation. These results confirmed that DEX‐CBX suppressed LPS‐induced inflammation and tumor cell proliferation.

### 2.3 Colonic Retention and Biodistribution of Oral DEX‐CBX

**Figure 3:**
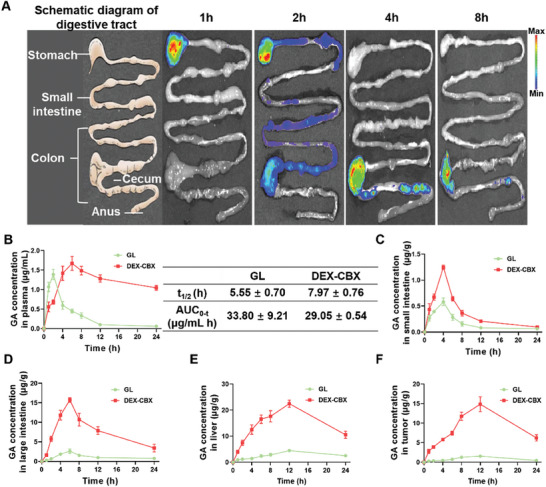
Gastrointestinal tract distribution and pharmacokinetics study of DEX‐CBX. A) Schematic illustrations of the mouse gastrointestinal tract and the ex vivo fluorescence images of the intestinesthe captured at 1, 2, 4, and 8 h after oral administration of DEX‐CBX. B) Pharmacokinetic results of oral GL or DEX‐CBX in orthotopic HCC bearing mice. C–F) Quantitative analysis of the GA biodistribution in small intestine, large intestine, liver, and tumor at different times after oral administration of free GL or DEX‐CBX in orthotopic HCC bearing mice. Data are shown as means ± SD (n = 3).

The distribution of dextranase in the digestive tract was evaluated through the enzyme linked immunosorbent assay (ELISA) test; dextranase is mainly present in the colon (Figure S8, Supporting Information). Additionally, most commensal microbiota, particularly LPS‐associated ones, reside in the colon. Therefore, we examined whether oral DEX‐CBX has a colon‐retentive property, which is critical for realizing continuous drug release and in situ gut microbiota modulation. The in vivo colonic retention of DEX‐CBX was investigated by visualizing the Cy5 signal in the isolated digestive tracts of BALB/c mice that were orally administered Cy5‐labeled DEX‐CBX. As shown in **Figure** **3**A, during the first 1–2 h post‐oral administration of DEX‐CBX, the red fluorescence of Cy5 was mainly detected in the stomach and small intestine. At 4 h post‐oral DEX‐CBX, the Cy5 fluorescence was mainly detected in the colon, especially in the cecum. The Cy5 fluorescence could remain strong in the cecum even at 8 h post‐oral DEX‐CBX. Such a long retention time in the colon would be beneficial for the continuous drug release and accumulation in the liver through the gut‐liver axis, as well as in situ gut microbiota modulation.

The GA concentrations in plasma, intestine, liver, and orthotopic HCC tissues were also quantitatively analyzed by HPLC, as the metabolites of CBX and GL were both GA.^[^ ^21^ ^]^ As shown in Figure 3B, an obvious level of GA could be detected in 1 h post‐oral free GL, and the GA concentration in plasma peaked at 2 h and then declined rapidly, with a terminal half‐life of 5.5 ± 0.70 h. In contrast, the GA concentration in plasma of the DEX‐CBX treated group was significantly lower than that of the free GL treated group within the first 4 h post‐treatment, which should be attributed to the low level of dextranase in the stomach and small intestine. While, the GA concentration in plasma of the DEX‐CBX treated group rapidly increased at 4 h post‐treatment, and higher drug concentrations in the plasma over that of the free GL treated group, with a reaching peak concentration at 6 h, followed by a slower decline, and a terminal half‐life of 33.80 ± 9.21 h. This was consistent with the intestinal fluorescence results, where the highest drug retention was seen in the large intestine, particularly the colon, at 4 h, with a decrease in fluorescence by 8 h. Further analysis showed that the GA concentration in the small intestine peaked at 4 h post‐treatment and then gradually decreased in both free GL and DEX‐CBX treated groups, which should be attributed to the limited retention time in the small intestine caused by gastrointestinal peristalsis (Figure 3C). As for the large intestine, the GA concentrations of free GL and DEX‐CBX treated groups both peaked at 6 h post‐administration and then gradually declined (Figure 3D). The GA concentrations in the liver and orthotopic HCC tissues peaked at 12 h post‐administration (Figure 3E,F). The GA content in the tumors of the DEX‐CBX treated group was much higher than that of the free GL treated group, which was ≈8.2 (at 6 h), 8.5 (at 12 h), and 10.0 (at 24 h) folds, respectively. In addition, the content of GA in the liver of the DEX‐CBX‐treated group was also much higher than that in the free GL‐treated group, which was ≈8.3 (at 6 h), 8.4 (at 12 h) and 12.7 (at 24 h) folds. As for DEX‐CBX, the GA concentrations in both plasma and liver reached their peak after DEX‐CBX reached the colon, confirming the synergistic effect of the colon‐specific drug activation and targeted accumulation to the liver via the gut‐liver axis of DEX‐CBX. As this rational design of orally administered drugs can improve the concentration of drugs in the liver and prolong their residence time in the liver, it should contribute to the treatment of HCC.

### 2.4 Oral DEX‐CBX for In Situ Modulation of Gut Microbiota

**Figure 4:**
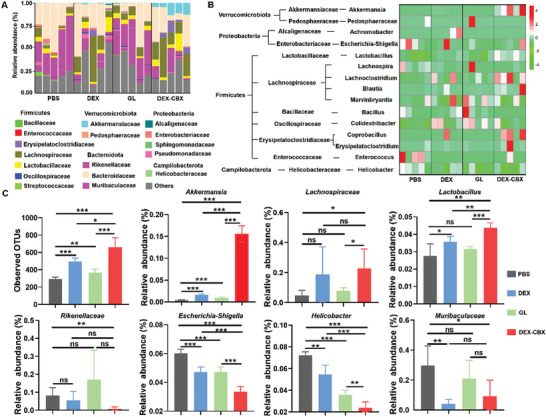
Distinct gut microbial communities are promoted by various treatments. A) Relative abundance of gut microbiome. Phylum‐ and family‐level taxonomy are presented as a percentage of total sequences, n = 5. B) Heatmap of the relative abundance of family‐level taxa (rows) for each mouse (columns). The abundance is shown as a relative percentage, n = 5. C) Estimation of microbial community observed OTU richness and relative abundance of select taxa, n = 5. Data are shown as means ± SD (n = 5). ^*^ *p* < 0.05, ^**^ *p* < 0.01, ^***^ *P* < 0.001; ns, no significance.

The gut microbiota dysbiosis has been demonstrated to be strongly correlated with liver disease, which contributes to intestinal barrier impairment, overgrowth of certain unfavorable microbiota as well as decrease of beneficial communities.^[^ ^22^ ^]^ The gut microbiota dysbiosis also orchestrates the tumor microenvironment of HCC and affects the anti‐tumor immune response through inflammation.^[^ ^23^ ^]^ As DEX‐CBX was fabricated with prebiotics (dextran) and anti‐inflammatory drug, changes in the gut microbiota of orthotopic H22 tumor‐bearing mice after receiving various treatments were investigated by analyzing the mouse fecal samples using 16S ribosomal RNA gene sequencing. Treatment with DEX, GL, or DEX‐CBX led to distinct clustering of the microbial community structure at the phylum and family level (**Figure** **4**A,B). Particularly, DEX‐CBX treatment significantly increased the overall richness of microbiota, and also significantly increased the relative abundance of *Akkermansia*, which is well‐known to strengthen systemic immune response (Figure 4C).^[^ ^24^ ^]^ Moreover, an increase in the relative abundance of *Lachnospiraceae* and *Lactobacillus* was also observed in DEX‐CBX treated groups. As both *Lachnospiraceae* and *Lactobacillus*, major producers of short‐chain fatty acids (SCFAs),^[^ ^25^ ^]^ were observed in the DEX‐CBX treated groups. This enhancement of these microbiota could potentially benefit T‐cell immunity in HCC management. In contrast, limited changes in the relative abundance of these microbiota were observed in the DEX and GL‐treated groups. Remarkably, a noticeable decrease in the relative abundance of bacterial genera associated with LPS production, including *Rikenellaceae*, *Escherichia‐Shigella*, and *Helicobacter* after DEX‐CBX treatment, which has been proven to be interrelated with microbial‐dependent inflammation and treatment resistance.^[^ ^26^ ^]^ Conversely, DEX and GL treatments showed mild modulations on these microbiota. The LPS level in the liver was also detected after various treatments. Consistent with the reduction in LPS‐associated microbiota, DEX‐CBX treatment significantly reduced the LPS level in the liver, which should be beneficial for alleviating hepatic inflammation caused by LPS (Figure S9, Supporting Information). In the treatment of orthotopic HCC, the single use of DEX or GL only achieved a limited positive effect on modulating the gut microbiota. For DEX, this limitation should be attributed to its short intestinal retention time.^[^ ^27^ ^]^ In aqueous solution, branched DEX mainly exists in the form of free molecules, which makes it difficult to form nanoparticles or gel with high strength, resulting in limited colon retention. As for GL, the main reason for its weak gut microbiota modulation was its low bioavailability. GL mainly relied on inflammation relief to modulate the gut microbiota. As shown in Figure 3, the GA concentration in the intestine and liver was low and quickly decreased. Therefore, the efficacy of inflammation modulation and HCC was limited, resulting in only mild modulation of the gut microbiota. In contrast, DEX‐CBX can self‐assemble into nanoparticles due to its amphiphilic structure (Figure 1C), achieving persistent colonic retention.^[^ ^28^ ^]^ Thus, DEX‐CBX treatment led to enhanced GA accumulation in HCC, inhibition of HCC, and relief of hepatic inflammation. This effect was further supported by prolonged colonic retention of DEX, contributing to better modulation of the gut microbiota. These results indicated that DEX‐CBX treatment not only suppresses LPS‐induced inflammation in the liver but also reduces LPS production by in situ modulation of the gut microbiota, which should be beneficial for HCC treatment by addressing both the symptoms and root cause of LPS‐induced hepatic inflammation.

### 2.5 In Vivo Antitumor Efficacy of DEX‐CBX

**Figure 5:**
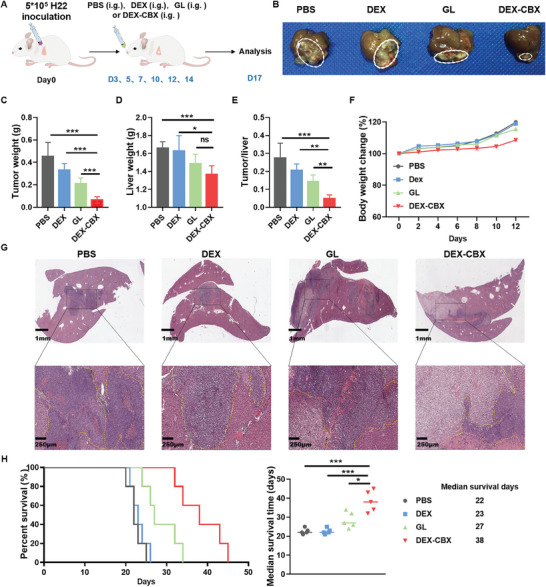
Tumor inhibition in orthotopic H22 tumor model. A) Treatment regimen. Representative photos of livers B), tumor weight C), liver weight D), tumor/liver weight ratios E), and body weight changes F) after mice received different treatments. G) H&E staining images of the liver of mice after receiving different treatments. H) Overall survival time of orthotopic H22 tumor‐bearing mice after receiving different treatments. Data are shown as means ± SD (n = 5). ^*^ *p* < 0.05, ^**^ *p* < 0.01, ^***^ *p* < 0.001; ns, no significance.

Orthotopic HCC‐bearing mice were used for treatment evaluation. Three days post‐tumor inoculation, the mice were randomly divided into four groups to receive different treatments: PBS (i.g.), DEX (i.g.), GL (i.g.), and DEX‐CBX (i.g.). The treatments were repeated six times (**Figure** **5**A). Fourteen days after the first treatment, the mice were sacrificed and the tumors were separated, photographed, and weighed. Liver weights and tumor‐to‐liver weight ratios were also recorded. As shown in Figure 5B,C, large tumor tissue could be observed on the liver, and the average tumor mass in PBS treated group was ≈0.45 ± 0.13 g. DEX treatment did not exhibit an obvious therapeutic effect compared with PBS treatment, as the final average tumor mass in the DEX‐treated group (0.33 ± 0.06 g) did not show a significant difference compared with that in the PBS‐treated group. Free GL showed a modest tumor inhibitory effect, with a final average tumor mass of 0.21 ± 0.05 g. In contrast, DEX‐CBX treatment significantly inhibited tumor growth, resulting in the lowest average tumor mass (0.07 ± 0.03 g) after the treatment. The liver weights of the DEX‐CBX‐treated groups were lower than those of the other treatment groups (Figure 5D). The tumor suppression rate of the DEX‐CBX‐treated group was 85.4%, which was higher compared with that achieved by the other treatments (26.6% in DEX‐treated groups and 53.3% in free GL‐treated groups). Additionally, the ratio of tumor to liver of DEX‐CBX treated group was lower compared with that of the other groups (Figure 5E). Further analysis of histological changes in tumors demonstrated that DEX‐CBX treatment caused larger areas of necrosis, characterized by shrinkage of the nucleus, compared with the other groups, which also proved that DEX‐CBX treatment significantly suppressed HCC growth (Figure 5G). No notable changes in body weight were observed during the treatment (Figure 5F). In addition, DEX‐CBX treatment significantly prolonged the median survival time of mice, which was ≈1.7 times longer than that of the PBS treatment, 1.7 times longer than that of the DEX, and 1.4 times longer than that of the free GL (Figure 5H). Safety profiles and antitumor activities were further evaluated using histological and blood chemistry analyses. As shown in Figure S10 (Supporting Information), no notable damage was observed in the HE analysis of the major organs of the mice, indicating that the treatment with DEX‐CBX did not cause any significant changes in liver or kidney function. These results indicated that DEX‐CBX treatment significantly reduced the HCC burden and was safe.

### 2.6 Tumor Immune Microenvironment Evaluation

**Figure 6:**
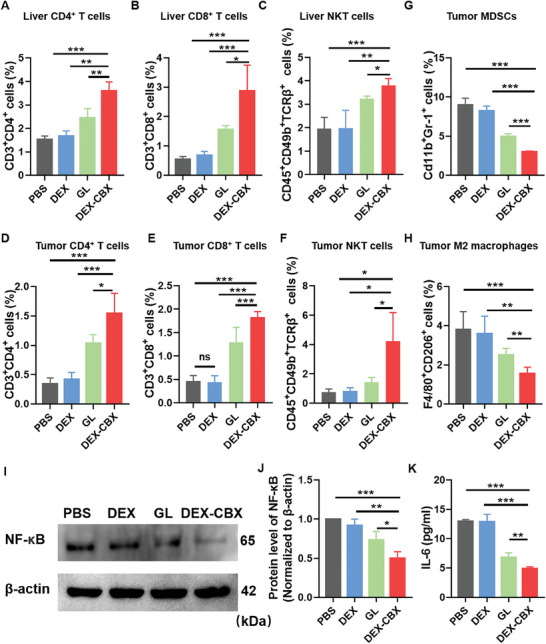
Changes in the immune microenvironment after various treatments. Proportions of liver‐infiltrating CD4^+^ T cells A), CD8^+^ T cells B), and NKT cells C) after mice received different treatments. (n = 5). Tumoral CD4^+^ T cells D), CD8^+^ T cells E), NKT cells F), MDSCs cells G), and M2 macrophages H) after mice received different treatments. (n = 5) I) Western blot images of NF‐κB expression in orthotopic HCC tissues after various treatments. J) Relative NF‐κB expressions in tumors of different groups compared to the PBS group; n = 3. K) The level of cytokines IL‐6 in tumors after mice received different treatments. (n = 4) Data are shown as means ± SD; ^*^ *p* < 0.05, ^**^ *p* < 0.01, ^***^ *p* < 0.001; ns, no significance.

Immune cells in the liver and tumors were further analyzed to investigate the immune responses after different treatments. As shown in **Figure** **6**A–F and Figures S11–S14 (Supporting Information), compared with the other treatments, DEX‐CBX treatment significantly increased the number of CD4^+^ T cells, CD8^+^ T cells, and Natural killer T (NKT) cells in the liver and orthotopic HCC tissues, especially NKT cells, which were demonstrated to actively protect the liver and contribute to antitumor activity and immunity via tumor‐related cytokines.^[^ ^27^ ^]^ Interferon‐gamma (IFN‐γ), a 1 type cytokine critical for both innate and adaptive immunity, was markedly increased in DEX‐CBX treatment compared with the other treatment, indicating that the recruited NKT and T cells were activated and exhibited antitumor effects (Figure S15, Supporting Information). DEX‐CBX treatment decreased the ratio of M2‐type macrophages to MDSCs (Figure 6G,H). DEX and free GL treatments had a weak effect on these changes. The expression of NF‐κB, a critical modulator of LPS‐induced inflammation, was also evaluated by western blot analysis. As shown in Figure 6I,J, DEX‐CBX treatment resulted in the strongest downregulation of NF‐κB expression. In addition, the IL‐6 levels decreased after DEX‐CBX treatment, indicating effective inflammation modulation by DEX‐CBX treatment. These results validated the improved tumor immunosuppressive microenvironment and stronger antitumor immune responses induced by DEX‐CBX treatment.

## 3 Discussion

The complicated crosstalk between the gut microbiota and the host immune system is the cause of numerous non‐infectious human diseases, such as autoimmune diseases, allergies, and cancer. These interactions hinge on the pro‐ and anti‐inflammatory responses caused by the gut microbiota. Recently, the gut‐liver axis has attracted extensive attention owing to its critical role in the progression of hepatic diseases.^[^ ^28^ ^]^ Toll‐like receptors (TLR), especially TLR4, play a crucial role in hepatic diseases by recognizing exogenous pathogen‐related molecular patterns and endogenous damage‐related molecular patterns, thereby triggering inflammatory immune responses.^[^ ^29^ ^]^ Aberrant activation of the TLR4 pathway has been extensively implicated in HCC progression and treatment resistance.^[^ ^30^ ^]^ The increased permeability of the intestinal wall caused by HCC leads to increased translocation of intestinal bacteria and elevated levels of hepatic LPS. Elevated levels of LPS perpetually activate TLR4 in hepatic macrophages and astrocytes initiating the NF‐κB signaling pathway, fostering cytokine secretion such as IL‐6, and culminating in liver inflammation and oxidative damage.^[^ ^31^ ^]^ IL‐6, a pivotal cytokine in HCC progression, orchestrates a spectrum of cellular responses in hepatocytes, Kupffer cells, and hepatic stellate cells, promoting the oncogenesis, progression, and metastasis of HCC.^[^ ^32^ ^]^ As an inflammation‐related cancer, HCC progression is associated with inflammation severity. Therefore, LPS‐induced inflammation is an attractive therapeutic target for preventing and treating HCC.

For LPS‐induced inflammation modulation, two schemes can be adopted: 1) using probiotics or antibiotics to inhibit intestinal bacterial translocation to inhibit LPS‐mediated TLR4 activation, and 2) directly inhibiting the activation of LPS‐mediated TLR4.^[^ ^33^ ^]^ Studies have shown that probiotics and antibiotics can reduce liver injury caused by TLR4 activation‐induced hepatitis. However, the transmission of probiotics faces the problems of low efficiency and unclear mechanisms. The use of antibiotics for treatment faces difficulty in achieving the regulation of specific bacteria, and due to the short duration of the treatment effect, long‐term use of antibiotics is necessary. Although long‐term use of antibiotics may change the balance of intestinal bacteria, it may also lead to dangerous side effects and complications, such as infection caused by the overgrowth of antibiotic‐resistant bacteria, malabsorption, and antibiotic‐related diarrhea.^[^ ^34^ ^]^ At present, few drugs directly inhibit TLR4 activation. Although the developed TAk‐242, E5564, and CRX‐526 showed the ability to inhibit the LPS‐induced inflammatory response and improve the survival rate of a sepsis model,^[^ ^35^ ^]^ there are no studies on the treatment of these drugs in chronic liver disease or HCC.

In this study, we employ a Trojan‐horse nanomedicine (DEX‐CBX) to simultaneously address both the symptoms and root causes of LPS‐induced immunosuppression in HCC immunotherapy. After oral administration, DEX‐CBX underwent activation by gut bacteria‐secreted dextranase and esterase, releasing GA, which subsequently accumulated in the liver via the gut‐liver axis. This process led to the inhibition of LPS‐TLR4‐induced inflammation. Furthermore, DEX‐CBX effectively modulated commensal microbiota in situ, reducing LPS production. The colonic drug release mechanism synergized with gut‐liver axis transport, facilitating enhanced and precise delivery of immune modulators to the liver for HCC immunotherapy. Consequently, this approach resulted in increased accumulation of CD8^+^ T and NKT cell and suppression of HCC. The proposed design presented a new strategy for precisely modulating gut microbiota and hepatic inflammation to reinvigorate the immune response for HCC cancer immunotherapy.

## 4 Experimental Section

### Materials and Characterization

^1^H NMR spectra were obtained using a Bruker AV‐500 or Bruker AV‐300 NMR spectrometer. Drug release and biodistribution assays were carried out with high‐performance liquid chromatography equipped (HPLC, Elite Analytical Instrument; Dalian, China) with an ultraviolet detection with a C18 chromatographic column. DLS was used to characterize the size of the self‐assembled nano micelles on the Malvern Zetasizer instrument (Nano‐ZS90). TEM images were obtained using a JEOL JEM‐1011 microscope (Tokyo, Japan). Histological alterations were observed using an optical microscope (Nikon Eclipse Ti, Optical Apparatus Co., Ardmore, PA, USA). All immunofluorescent and cellular uptake slides were imaged using a confocal laser scanning microscope (CLSM, Carl Zeiss LSM 700, Germany).

Dextran (DEX) with an average molecular weight of 40,000 Da was provided by Shanghai Aladdin Biochemical Technology Co., Ltd. Carbenoxolone was obtained from Nanjing Kangmanlin Chemical Industry Co., Ltd. Glycyrrhizic acid was obtained from Nanjing Kangmanlin Chemical Industry Co., Ltd. The murine HCC cell line (H22) was purchased from the BeNa Culture Collection. Esterase and dextranase were purchased from Shanghai Yuanye Biotechnology Co., Ltd. IFN‐γ, IL‐6, IL‐1β, and TNF‐α ELISA kits were purchased from Anoric Biotechnology. Tech. LPS ELISA kit was purchased from Shanghai Enzyme‐linked Biotechnology Co., Ltd. Alanine transaminase (ATL), aspartate transaminase (AST), urea nitrogen (BUN), and creatinine (CRE) was obtained from Maker Biotechnology. *N*,*N*'‐Diisopropylcarbodiimide (DIC) was obtained from Beijing J&K Co., Ltd. 4‐Dimethylaminopyridine (DMAP) was provided by Adamas Reagent, Co., Ltd. Dimethyl sulfoxide (DMSO) was purchased from Anhui Zisheng Technology Co., Ltd. HEK‐Blue hTLR4 cell lines, was purchased from InvivoGen. All antibodies and elisa kits were provided by BD Biosciences, BioLegend or Invitrogen, as shown in Table S1 (Supporting Information).

### Preparation of DEX‐CBX Conjugate

A dextran‐carbenoxolone conjugate (DEX‐CBX) was prepared by esterification of the hydroxyl group in DEX with the carboxyl group of succinic acid in CBX. Briefly, DEX (1000 mg, 0.025 mmol), CBX (250 mg, 0.44 mmol), and DMAP (53.5 mg, 0.44 mmol) were dispersed in anhydrous DMSO. Then, the DMSO solution of DIC (82.5 mg, 0.66 mmol) was added into the above mixture under stirring. After 36 h, the mixture was dialyzed directly against sterile water for 48 h. The final product was obtained by lyophilization.

The drug loading content (DLC) of DEX‐CBX was determined using an HPLC system. 10 mg DEX was hydrolyzed with 2 mLNaOH aqueous solution (1 m) for 1, 2, 4, 8, and 12 h, then, 2 mL 1.2 m phosphoric acid was added. Subsequently, 5 mL methanol was added, and the sample was dried with a nitrogen‐blowing instrument. A total 9 mL acetonitrile was added to extract GA as CBX could be converted to GA in the hydrolysis of NaOH, the extraction solution was set to a volume of 10 mL. The content of GA was detected, and the content of CBX was calculated by the ratio of molecular weight between CBX and GA.

As for Cy5‐COOH labeled DEX‐CBX, Cy5‐COOH (10.0 mg), DIC (5.0 mg), and DMAP (7.0 mg) were added to a DMSO solution of DEX‐CBX (90.0 mg), and the mixture was stirred for 12 h following dialyzing against sterile water for 72 h in the dark. A blue powder was obtained after lyophilization.

### Solution Behavior of DEX‐CBX Conjugate

The nano micelles of DEX‐CBX were formed by dispersion in phosphate‐buffered saline (PBS, 0.01 m, pH 7.4). The diameters of the samples were evaluated using a Malvern PANalytical Zetasizer Nano ZS90 instrument. To obtain TEM images, an aqueous solution of DEX‐CBX was dropped onto a copper net and left alone until the water evaporated completely. The TEM was performed using a JEOL‐JEM‐1011 TEM at an accelerating voltage of 100 kV.

### In Vitro Drug Release

The drug‐release profile was investigated using dialysis. Because GA is essentially a pharmacological formulation of glycyrrhizin drugs, GA in the release solution was detected using HPLC to quantitatively study the release profile of DEX‐CBX. Briefly, dialysis bags each containing 5 mL DEX‐CBX solution (0.12 mg CBX/mL) were separately immersed in 45 mL different release buffer under shaking (90 rpm) at 37 °C. The release buffer included pH 1.2 NaCl‐HCl buffer (0.2 m), pH 7.4 PBS (0.01 m), pH 7.4 PBS (0.01 m) with 40 U mL^−1^ dextranase and pH 7.4 PBS (0.01 m) with 40 U mL^−1^ dextranase and 40 U mL^−1^ esterase. All the release buffers contained 0.2% Tween 80. At predetermined time points (1, 2, 4, 8, 12, 24, and 48 h), a 5.0 mL release solution was collected, and fresh buffer (5.0 mL) was added. The obtained release solution was added 2 mL 1 m NaOH aqueous solution, and incubated for 4 h to convert CBX to GA. After that, 2 mL 1.2 m phosphoric acid was added. Subsequently, these release samples were freeze‐dried, and the GA in this sample was extracted with acetonitrile after removing salt with centrifugation for further testing. The release tests were repeated three times under the same conditions. The HPLC mobile phase consisted of water and acetonitrile (20/80, V/V) at a flow rate of 0.8 mL min^−1^ and a wavelength of 254 nm.

### Cell Lines and Animal Declaration

H22 cells and RAW 264.7 cells were cultured in Dulbecco's modified Eagle's medium (DMEM) (supplementing 10% fetal bovine serum, 50 U mL^−1^ penicillin, and 50 U mL^−1^ streptomycin), and incubated at 37 °C with 5% CO_2_. HEK‐Blue hTLR4 cells were cultured in DMEM (supplementing 10% fetal bovine serum, 50 U mL^−1^ penicillin, and 50 U mL^−1^ streptomycin), and incubated at 37 °C with 5% CO_2_.

BALB/c mice (female, 8 weeks old, 18−20 g) were provided by Beijing Vital River Laboratory Animal Technology Co., Ltd. (China). Kunming mice (female, 8 weeks old, 18−20 g) were provided by the Laboratory Animal Center of Jilin University (Changchun, China). All animal experiments were approved by the Animal Care and Use Committee of Jilin University (approval number: 2021–173) and performed according to the guidelines of the Laboratory Animals of Jilin University.

### Cytotoxicity Assays In Vitro

The cytotoxicity of free GL, DEX‐CBX, and DEX‐CBX with esterase and dextranase was detected using a methyl thiazolyl tetrazolium (MTT) assay. The murine H22 hepatocellular cells were inoculated into 96‐well plates (8000 cells per well) and cultured overnight with 200 µL DMEM. Then free GL, DEX‐CBX, and DEX‐CBX with enzymes (40 U mL^−1^ esterase and 40 U mL^−1^ dextranase) (all these formulations containing 0‒500 µg CBX/mL) were added following further incubating for another 24 or 48 h. At determined time points, 20 µL MTT reagents were added to the plates for 4 h of incubation. After that, the culture medium was completely removed, and 150 µL DMSO was added to each well. The absorbance of each well was measured at 490 nm using a microplate reader. The cell viability (%) was calculated by comparing the absorbance value of the sample with that of the control.

### Evaluation of the Inhibitory Effect of DEX‐CBX on TLR4 Activation Induced by LPS

2 × 10^4^ RAW 264.7 cells were seeded in a 48‐well plate with 400 µL culture medium. Stimulation with LPS (100 ng mL^−1^) to activate TLR4. After overnight incubation, the culture medium was replaced with 360 µL fresh medium containing PBS (40 µL) (negative control), sterile water (20 µL), and LPS (20 µL of 2000 ng LPS /mL solution) (positive control), LPS with DEX, LPS with GL, or LPS with DEX‐CBX (20 µL of 500−2000 µg CBX/mL solution containing 40 U mL^−1^ dextranase) for another 24 h incubation. Subsequently, the cell supernatant was extracted and stored at −80 °C before detection. The activation of TLR4 was evaluated by measuring the level of IL‐6 in the cell supernatant.^[^ ^36^ ^]^ The inhibitory effects were evaluated by comparing the IL‐6 content of the samples from the treated groups and that of the positive control.

The activation of TLR4 was evaluated with HEK‐Blue hTLR 4 cells according to the previous report.^[^ ^19^ ^]^ HEK‐Blue hTLR 4 cells were inoculated in plated into a 96‐well plate at a density of 2.5×10^4^ cells per well. Then the cells were treated with PBS, LPS (250 ng mL^−1^), LPS with DEX, LPS with GL, and LPS with DEX‐CBX (50 µg CBX/mL, with/without dextranase). After 24 h incubation, the supernatants were collected and mixed with Quanti‐Blue. TLR activation related to SEAP activity was determined by measuring the OD value at 620 nm of Quanti‐Blue.

### Digestive Tract Distribution of DEX‐CBX After Oral Administration

To investigate the digestive tract distribution of DEX‐CBX after oral administration, BALB/c mice received 35 mg kg^−1^ of Cy5‐DEX‐CBX (i.g.). At each time point (1, 2, 4, and 8 h), three mice were sacrificed. The stomach, small intestine, and colon were then excised. The distribution of DEX‐CBX in the digestive tract was visualized using a Davinch‐Invivo HR imaging system (Davinch, Korea) with an excitation wavelength of 650 nm and an emission wavelength of 680 nm.

### Biodistribution Study of DEX‐CBX

To establish an orthotopic HCC model, 5 × 10^5^ H22 cells were injected into the abdomen of Kunming mice to accelerate the growth of H22 cells. Ascites were extracted from Kunming mice 5 d after H22 cell injection. Finally, the H22 cells were collected, washed with PBS, and diluted to a concentration of 2 ×10^7^ cells per mL for further construction of the orthotopic HCC model.

The biodistributions of GL and DEX‐CBX in major organs and tumors were assessed in orthotopic H22 tumor‐bearing mice. H22 cells (5×10^5^, 25 µL) were injected slowly into the right hepatic lobe and the abdomen was closed with the successful establishment of the orthotopic H22 tumor model. After 7 d of establishing the orthotopic H22 tumor model, mice received free GL (57.8 mg kg^−1^, i.g., equal GA amount with DEX‐CBX) and DEX‐CBX (40 mg CBX/kg, i.g.). In the ruddy liver tissue, the hard white tissue was identified as tumor tissue. The major organs (large intestine, small intestine, and liver) and tumors were excised at predetermined time points (1, 2, 4, 8, 12, and 24 h), weighed, ground, and homogenized. The total drug was extracted using acetonitrile. Finally, the GA content was detected by HPLC according to a previously reported method.^[^ ^37^ ^]^

### Orthotopic HCC Therapy

To establish an orthotopic H22 tumor model, female mice were anesthetized and disinfected, and the liver was exposed through an incision of 1 cm in the middle of the abdomen. Afterward, 5×10^5^ cells were injected into the left lobe of the liver, and the pinhole was immediately coagulated with an electric knife. Subsequently, the incision was sutured and disinfected. After 3 d of tumor inoculation, the mice were randomly divided into four groups and treated with PBS (i.g.), DEX 208.4 mg kg^−1^ (equal dextran amount to DEX‐CBX treatment, i.g.), GL 57.8 mg kg^−1^ (equal GA amount to DEX‐CBX treatment, i.g.), and DEX‐CBX (40 mg kg^−1^, i.g.) on days 3, 5, 7, 10, 12, and 14. Body weights were monitored every 2 d. On day 17, all mice were euthanized and tumor and liver weights were excised and recorded. The tumor suppression rate (TSR%) was calculated as follows: TSR% = [(A_c_ − A_x_)/A_c_] × 100%, where A_c_ and A_x_ are the mean tumor weight of the PBS group and the treatment groups (DEX, GL, or DEX‐CBX), respectively.

For the mouse survival study, the mice received the same treatment schedule as that for the tumor inhibition experiment, and mouse deaths were recorded until all mice died.

### Immunohistochemical Analysis

After 17 d of starting the treatments, the excised liver tumors and major organs (heart, liver, spleen, lungs, and kidneys) were fixed in 4% buffered paraformaldehyde, embedded in paraffin, sliced 5 µm thickness, and stained with hematoxylin and eosin (H&E) to examine histological pathology. The histological changes were observed under a microscope.

### Hepatotoxicity and Nephrotoxicity Analysis

Peripheral blood was collected from mice treated with PBS, DEX, GL, or DEX‐CBX on day 17. Serum concentrations of alanine aminotransferase (ALT), aspartate aminotransferase (AST), blood urea nitrogen (BUN), and creatinine (CREA) were measured using specific kits, according to the manufacturer's instructions.

### Flow Cytometry Analysis

The tumors and livers of the PBS‐, DEX‐, GL‐, and DEX‐CBX‐treated groups were collected on day 17 (n = 5) and analyzed using flow cytometry to detect changes in immune cells. Tumors were cut into small pieces and digested using tumor dissociation buffer containing 0.8 mg mL^−1^ type IA collagenase, 0.1 mg mL^−1^ DNase I, and 0.1 mg mL^−1^ hyaluronidase at 37 °C for 1 h and homogenized with RPMI 1640 containing collagenase IV. The supernatants from the digested tumor tissues were collected, filtered, centrifuged, and resuspended. Single‐cell suspensions were stained with the fluorophore‐conjugated antibodies (Table S1, Supporting Information). The stained cells were washed in FACS buffer, and fixed in 4% paraformaldehyde, and flow cytometry analysis was performed on a BD FACS Celesta flow cytometer (BD Biosciences).

The livers were ground, filtered, and re‐suspended in RPMI 1640 medium. Red blood cell lysis buffer was used to lyse the erythrocytes. Finally, the cell suspensions were stained with fluorophore‐conjugated antibodies, and flow cytometry analysis was performed using a BD FACS Celesta flow cytometer (BD Biosciences).

### Enzyme‐Linked Immunosorbent Assay

To analyze cytokines in tumor tissues, tumors were harvested, homogenized in PBS, and centrifuged at 3000 g for 10 min. ELISA kits were used to test the levels of IFN‐γ, TNF‐α, IL‐1β, IL‐6, and LPS in the supernatants were detected using ELISA kits according to the manufacturer's instructions. First, 50 µL of standard samples of different concentrations were added to the standard well, and then 10 µL of the test sample was to the sample well followed by 40 µL of sample diluent. Subsequently, 100 µL of HRP‐conjugate reagent was added to the standard and sample wells and placed in a constant temperature incubator for 60 min. After washing the plate 5 times, add 50 µL of each substrate A and B, and incubate at 37 °C in the dark for 15 min. Finally, 50 µL of termination solution and measured OD values of each well at a wavelength of 450 nm were added.

### Statistical Analyses

All results are expressed as the means ± standard deviation (SD). The analysis of variance was performed using a two‐tailed student's *t*‐test or one‐way analysis of variance (ANOVA). For survival analyses, log‐rank tests were performed to determine the overall *P*‐values. All statistical analyses were performed using GraphPad Prism 8. *P* < 0.05 were indicated statistically significant (*P < 0.05, **P < 0.01, ***P < 0.001), and ns indicated not significant.

## Conflict of Interest

The authors declare no conflict of interest.

## Author Contributions

S.M., W.S., G.L., and G.W. designed the research. H.Y. and S.M. conducted experiments and analyzed data. S.M. and H.Y. wrote the draft of the manuscript. S.M., W.S., and G.W. revised the manuscript. J.H., X.S., and M.Y. participated in part of the experiments. All authors contributed and approved the final manuscript.

## Supporting information



Supporting Information

## Data Availability

The data that support the findings of this study are available from the corresponding author upon reasonable request.
