# Supplementary material for: Trojan‐Horse Strategy Targeting the Gut‐Liver Axis Modulates Gut Microbiome and Reshapes Microenvironment for Orthotopic Hepatocellular Carcinoma Therapy
Source: Adv Sci (Weinh). 2024 Oct 7;11(44):2310002. doi: 10.1002/advs.202310002 (PMC11600211; doi:10.1002/advs.202310002)
Supplement: Supplementary file 1 — Supporting Information [file ADVS-11-2310002-s001.docx]

Supporting Information for

**Trojan-horse Strategy Targeting the Gut-Liver Axis Modulates Gut Microbiome and Reshapes Microenvironment for Orthotopic Hepatocellular Carcinoma Therapy**

Haochen Yao^1,2^, Sheng Ma^3,4^*, Juanjuan Huang^2,5^, Xinghui Si^3,4^, Ming Yang^6^, Wantong Song^3,4^, Guoyue Lv^1^* and Guoqing Wang^2^*

^1^Hepatobiliary and Pancreatic Surgery Department, General Surgery Center, First Hospital of Jilin University, No.1 Xinmin Street, Changchun, 130021, Jilin, China

^2^Key Laboratory of Zoonosis, Chinese Ministry of Education, College of Basic Medical Sciences, Jilin University, Changchun, 130021, Jilin, China

^3^Key Laboratory of Polymer Ecomaterials, Changchun Institute of Applied Chemistry, Chinese Academy of Sciences, 5625 Renmin Road, Changchun, 130022, China

^4^Jilin Biomedical Polymers Engineering Laboratory, Changchun Institute of Applied Chemistry, 5625 Renmin Road, Changchun, 130022, China

^5^Department of Computational Mathematics, School of Mathematics, Jilin University, Changchun, China

^6^Department of Molecular Biology, College of Basic Medical Sciences, Jilin University, Changchun, China


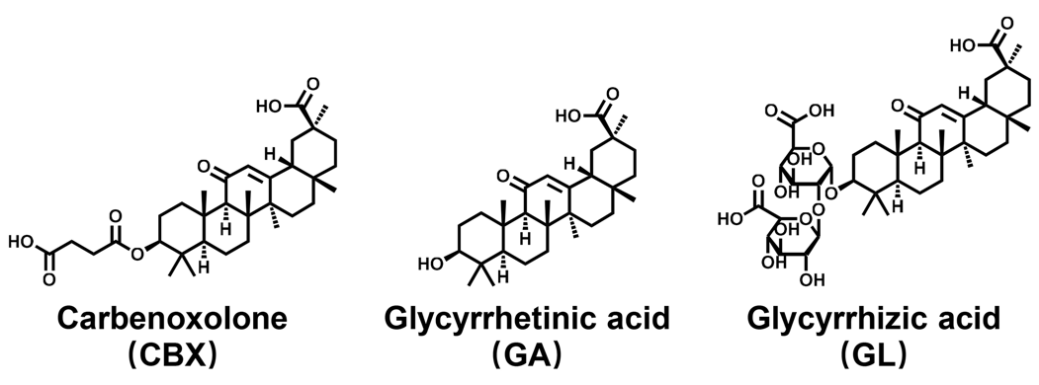


**Figure S1.** Structural formulations of carbenoxolone (CBX), glycyrrhetinic acid (GA) and glycyrrhizic acid (GL).


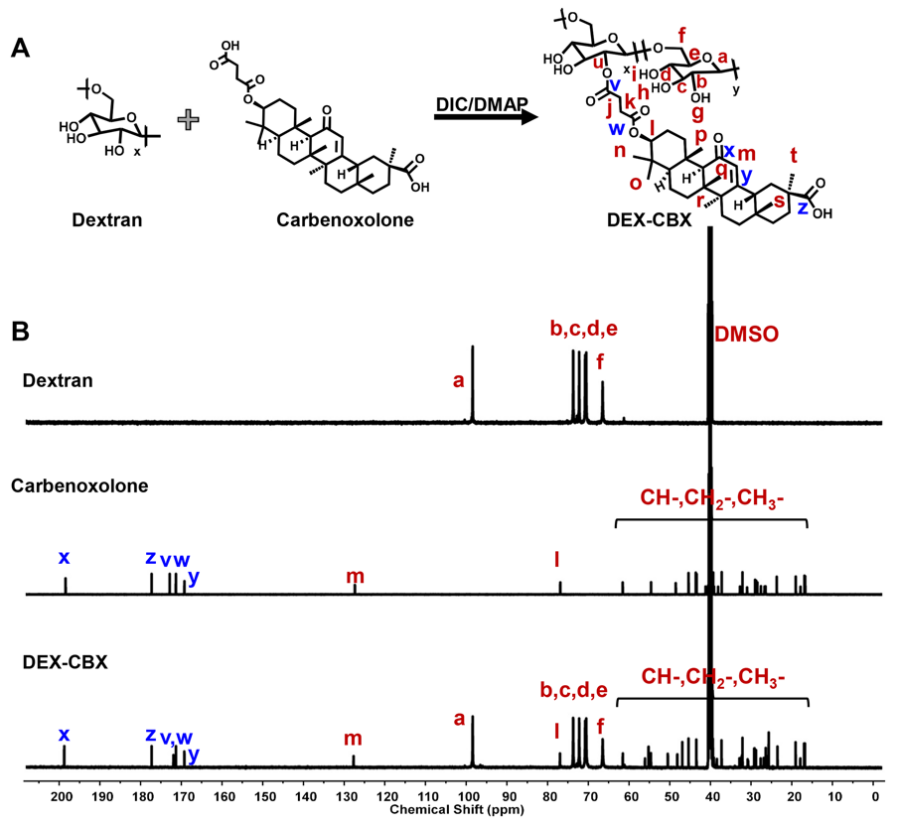


**Figure S2.** ^13^C NMR spectrum of DEX, Carbenoxolone, DEX-CBX in DMSO-d_6_.

**
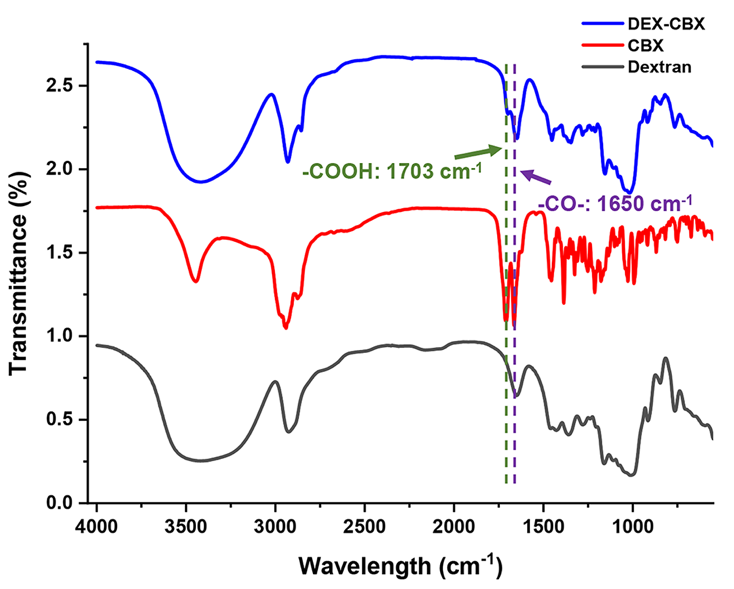
**

**Figure S3.** FT-IR spectrum of DEX-CBX, CBX and DEX.


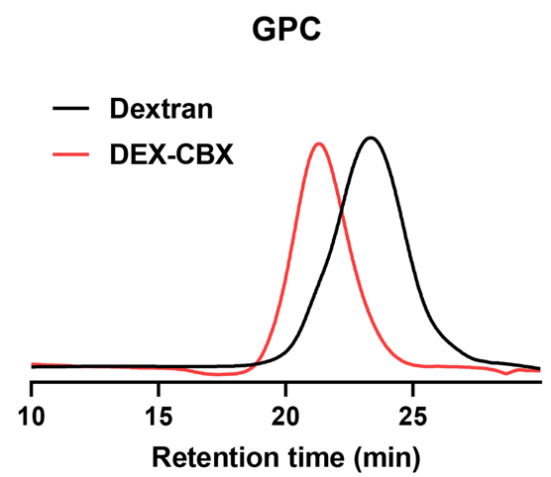


**Figure S4.** GPC curves of dextran and DEX-CBX.


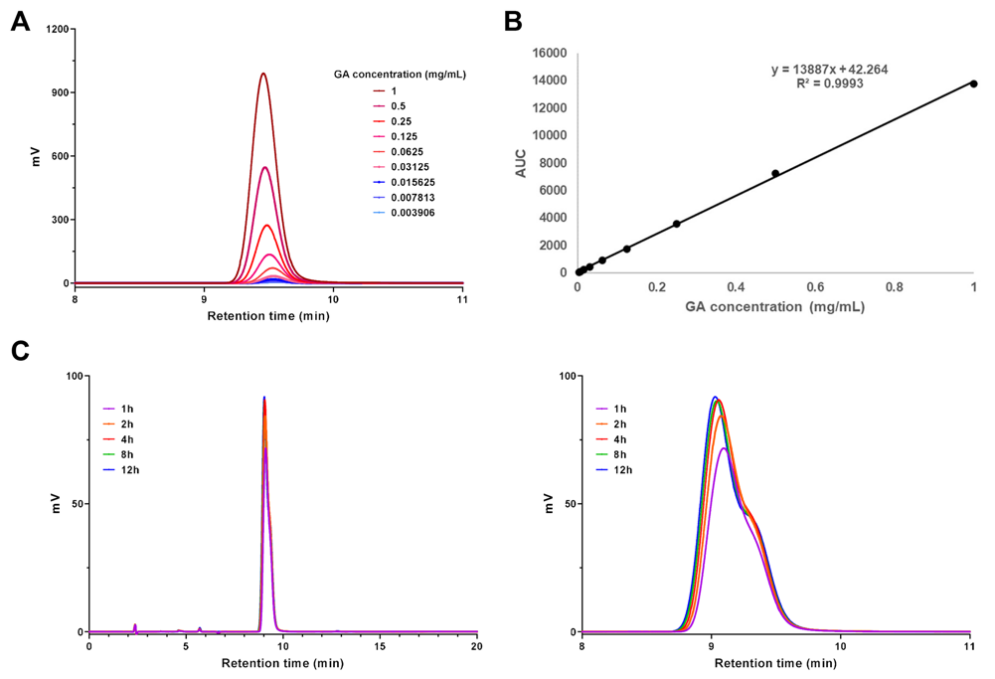


**Figure S5.** (A) HPLC analysis results of GA from 1-0.0039 mg/mL with water and acetonitrile (20/80, V/V) as mobile phase. (B) The linear relationship between HPLC peak area and GA concentration. (C) HPLC analysis the hydrolysis product of DEX-CBX after incubated with NaOH aqueous solution for different time.


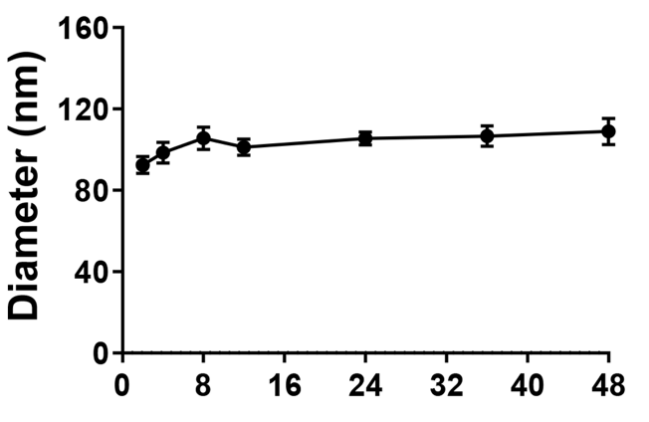


**Figure S6.** Stability of DEX-CBX in PBS. Data are shown as mean ± SD (n = 3).

**
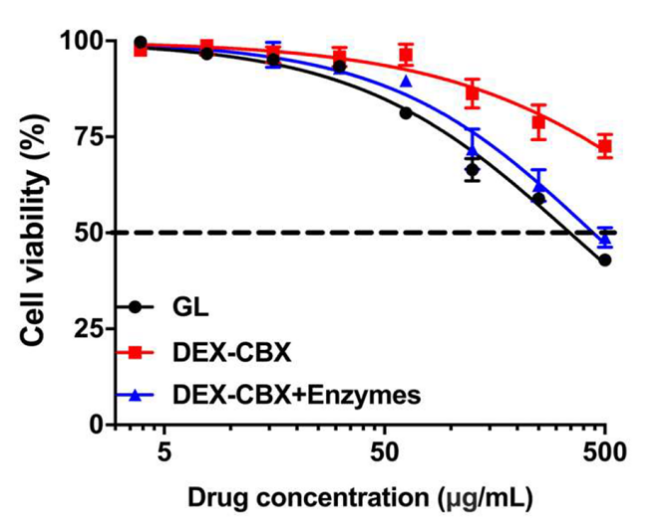
**

**Figure S7.** In vitro cytotoxicities of free [GL](https://www.sciencedirect.com/topics/biochemistry-genetics-and-molecular-biology/dexamethasone), DEX-CBX and DEX-CBX with Dextranase to H22 cells at different drug concentrations after incubation for 24 h.


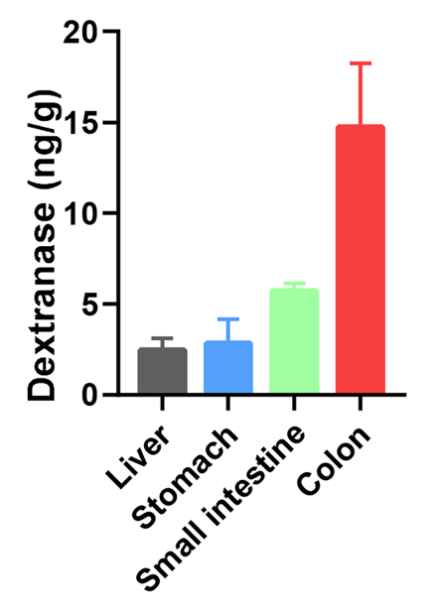


**Figure S8.** The content of dextranase in the liver and GI tract, data are shown as means ± SD (n = 4).


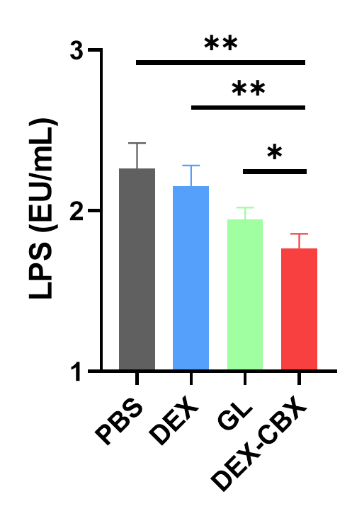


**Figure S9.** The content of LPS in the liver after various treatments, data are shown as means ± SD (n = 4).

**
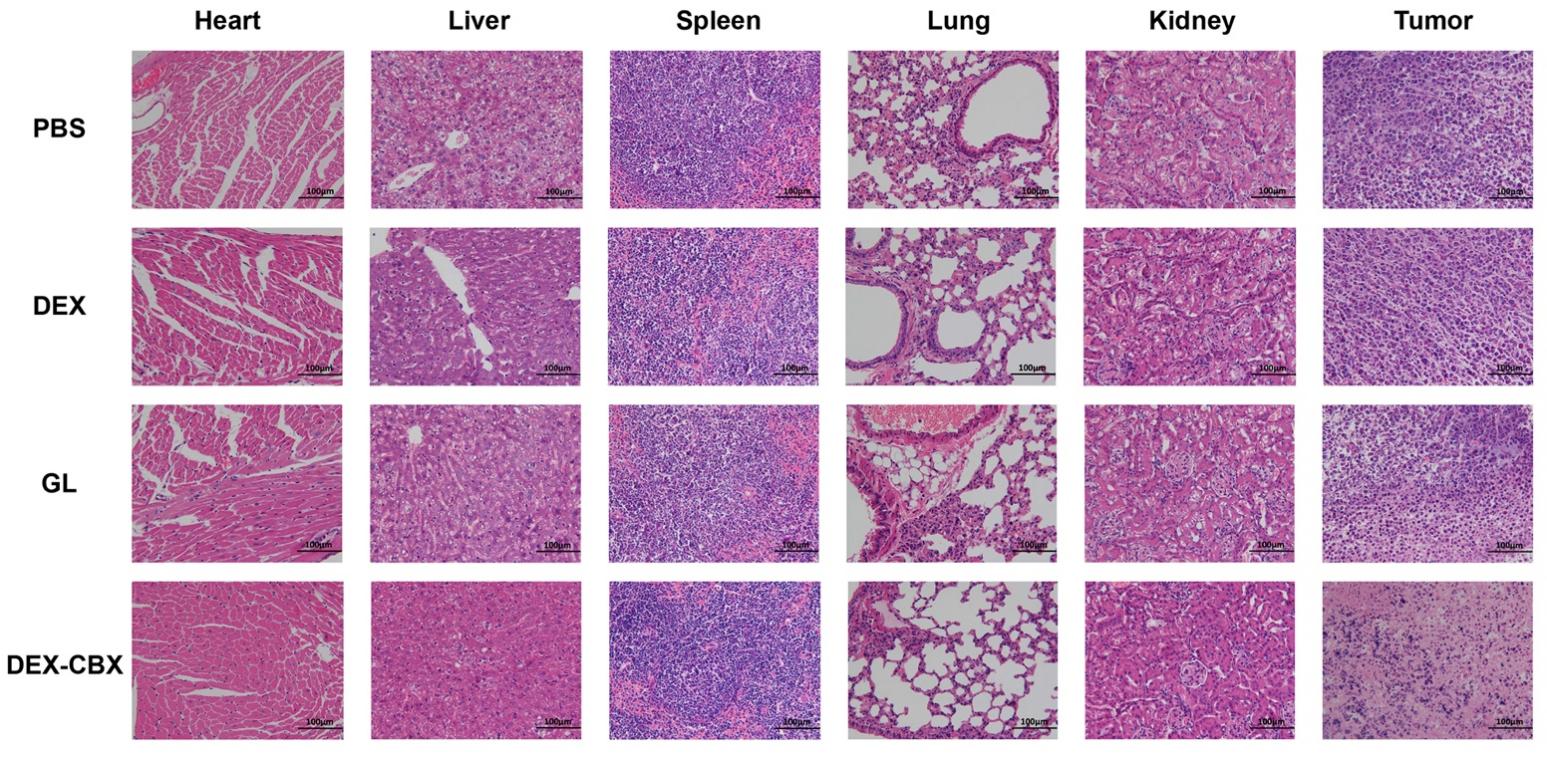
**

**Figure S10.** Histological analysis of different organs (heart, liver, spleen, lungs and kidneys) in primary H22 model after various treatments.


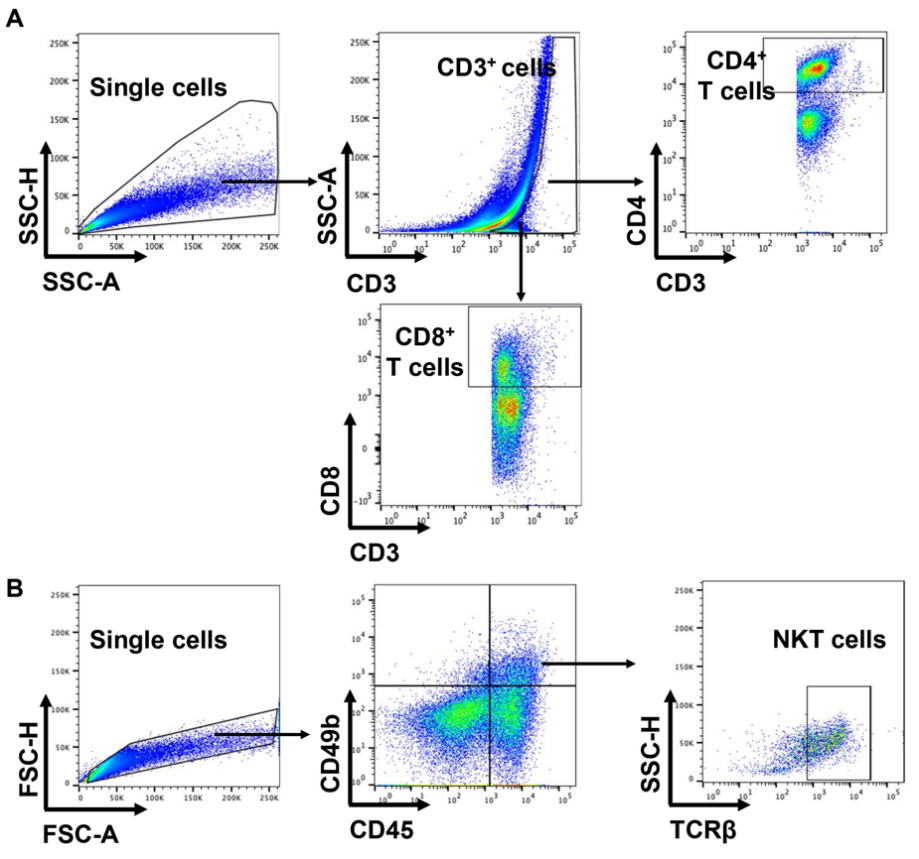


**Figure S11.** Gating strategies for CD4^+^ and CD8^+^ T cells in liver (A), and NKT cells (B) in liver.


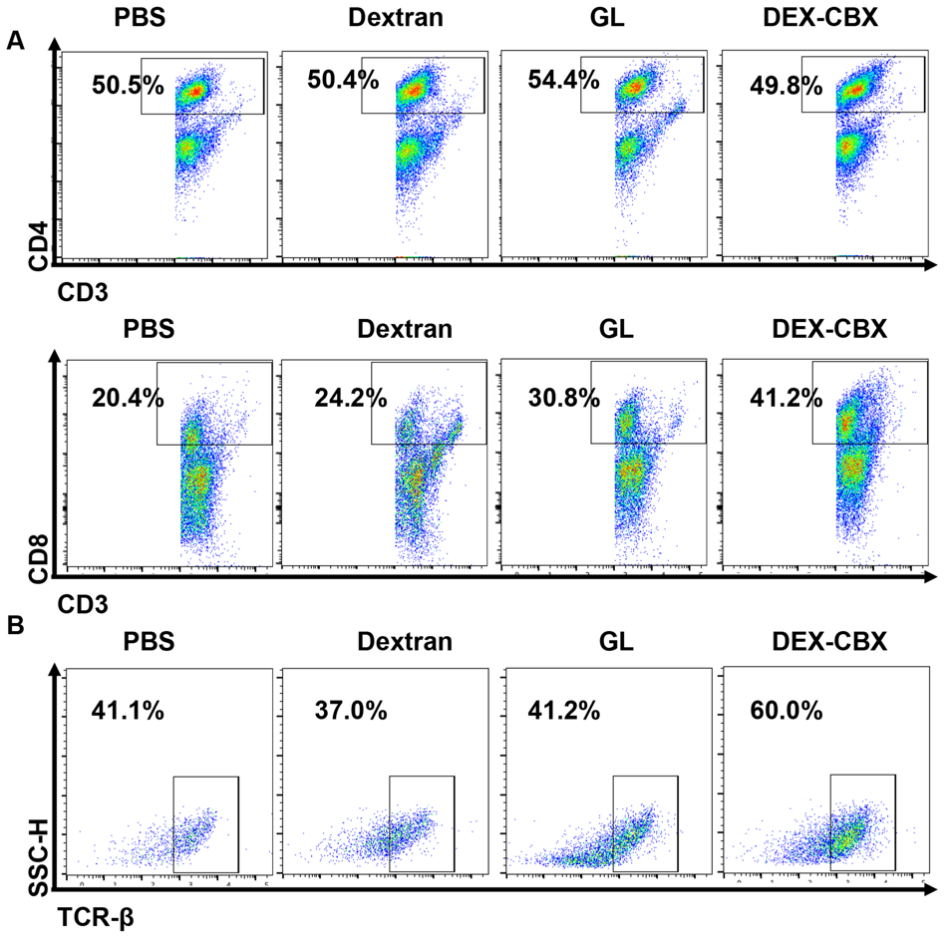


**Figure S12.** Representative flow cytometric analysis images CD4^+^ and CD8^+^ T cells (A), and NKT cells (B) in liver.


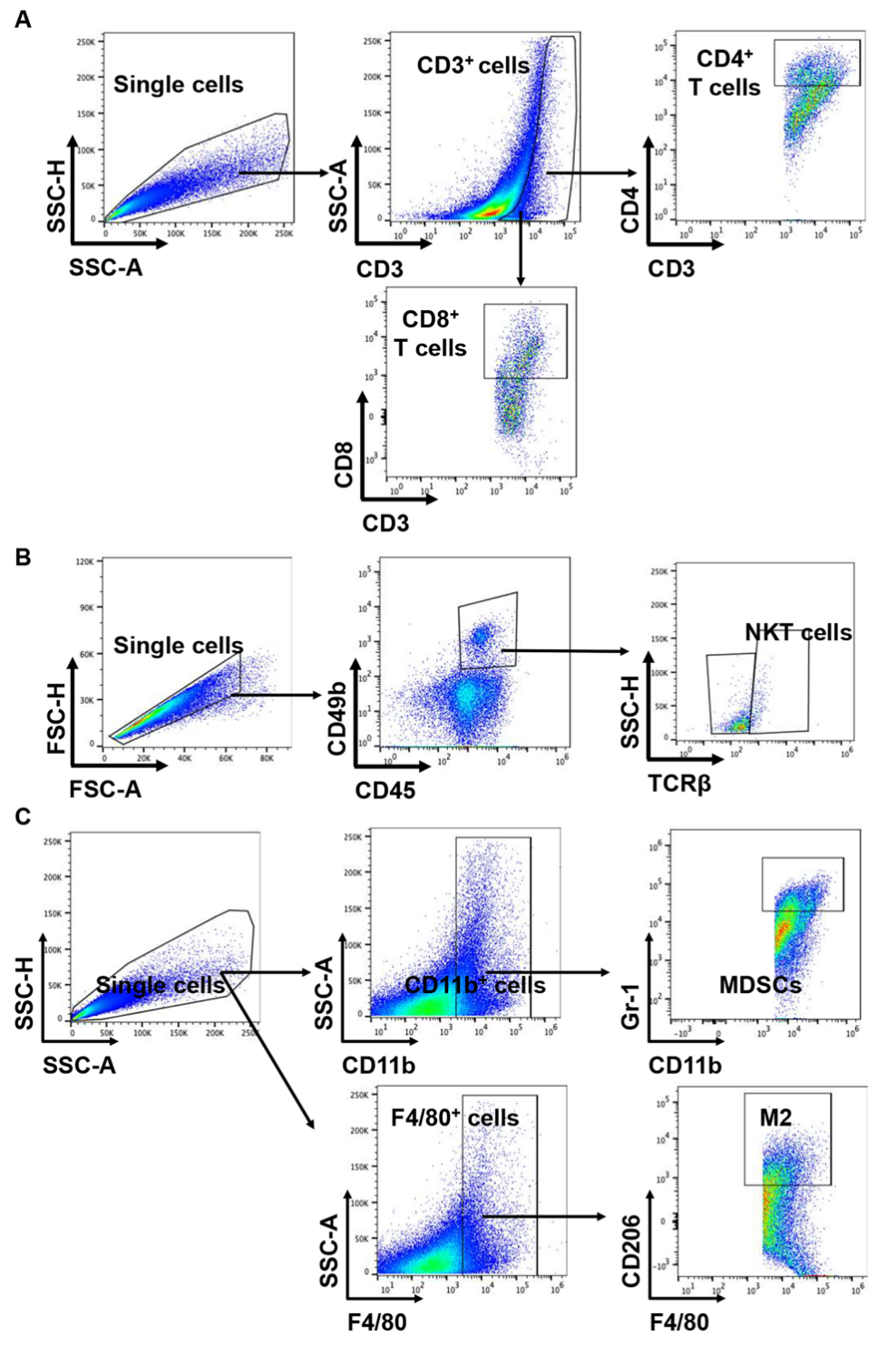


**Figure S13.** Gating strategies for CD4^+^and CD8^+^ T cells (A), NKT cells (B), and MDSCs and M2 macrophages (C) in tumors.

**
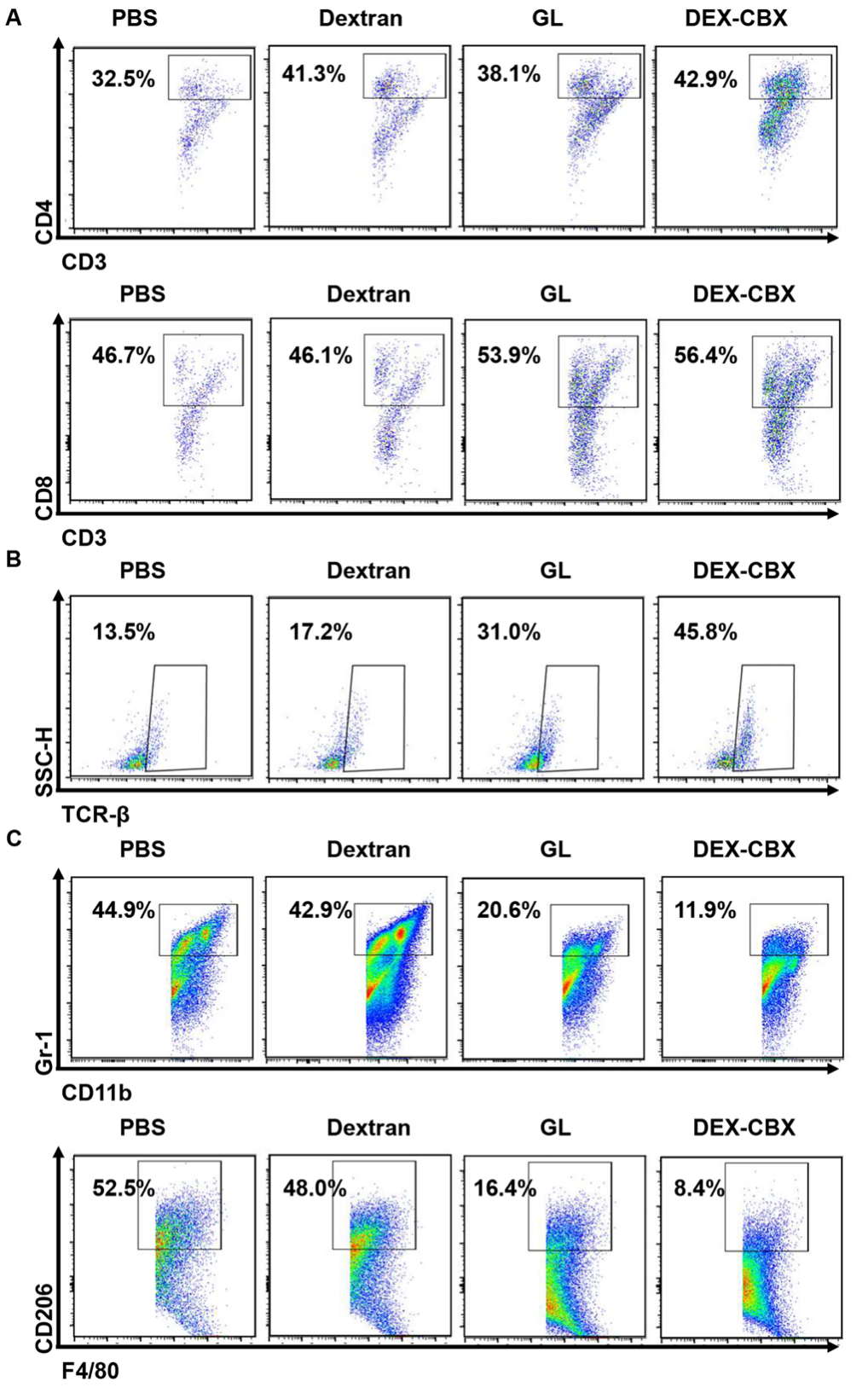
**

**Figure S14.** Representative flow cytometric analysis images of CD4^+^and CD8^+^ T cells (A), NKT cells (B), and MDSCs and M2 macrophages (C) in tumor.

**
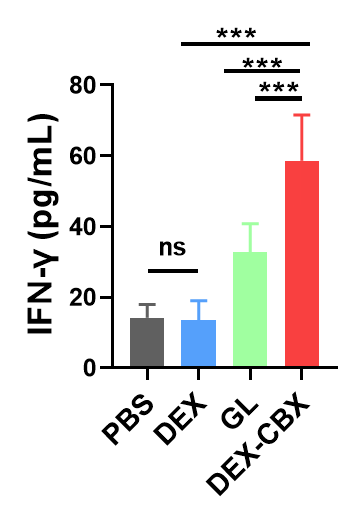
**

**Figure S15.** The IFN-γ content in tumor tissues after various treatments.

**Table S1. List of antibodies and ELISA kits used in this study**

| **Antibodies** | **Company** | **Catalog** | **Application** |
| --- | --- | --- | --- |
| FITC anti-mouse CD3 Antibody | BioLegend | 100204 | Flow cytometry |
| PE/Cy7 anti-mouse CD4 Antibody | BioLegend | 100422 | Flow cytometry |
| APC anti-mouse CD8a Antibody | BioLegend | 100712 | Flow cytometry |
| BV650 anti-mouse CD206 Antibody | BioLegend | 141723 | Flow cytometry |
| PerCP/Cy7 anti-mouse F4/80 Antibody | Biolegend | 123114 | Flow cytometry |
| FITC anti-mouse CD11b Antibody | BioLegend | 101205 | Flow cytometry |
| APC/Cy7 Anti-mouse CD45 Antibody | BioLegend | 103116 | Flow cytometry |
| PE Anti-mouse Gr-1 Antibody | Biolegend | 108408 | Flow cytometry |
| FITC Anti-mouse CD49b Antibody | Invitrogen | 12-5971-81 | Flow cytometry |
| APC Anti-mouse TCRβ Antibody | BD | 561080 | Flow cytometry |
| [Mouse IL-6 ELISA Kit](https://www.mlbio.cn/goods-95925.html) | Anoric Biotechnology. Tech | TAE-385 | ELISA |
| [Mouse IL-1β ELISA Kit](https://www.mlbio.cn/goods-95925.html) | Anoric Biotechnology. Tech | TAE-370 | ELISA |
| Mouse TNF-a ELISA kit | Anoric Biotechnology. Tech | TAE-569 | ELISA |
| Mouse IFN-γ ELISA kit | Anoric Biotechnology. Tech | TAE-366 | ELISA |
| Mouse LPS ELISA Kit | Shanghai Enzyme-linked Biotechnology Co., Ltd. | ml037221-1 | ELISA |
